# Achieving reproducibility in the innovation process

**DOI:** 10.12688/openreseurope.19408.1

**Published:** 2025-01-27

**Authors:** Maurice Whelan, Eann Patterson

**Affiliations:** 1 European Commission Joint Research Centre (JRC), Ispra, 21027, Italy; 2 School of Engineering, University of Liverpool, Liverpool, L69 3GH, UK

**Keywords:** Innovation, reproducibility, discovery, translation, application

## Abstract

Reproducibility is essential for innovation but is often hard to achieve in practice. One reason for this is a lack of appreciation of what needs to be reproduced and how in each phase of the innovation process. In the discovery phase, conclusions need to be reproduced through orthogonal investigation. In the translation phase, key attributes and outputs of derived products or processes should be reproducible by defining transferable specifications and protocols, whereas in the application phase, the goal is to achieve reproducible performance in real-world environments through appropriate quality assurance systems.

## Plain language summary

Reproducibility is essential for innovation but is often hard to achieve in practice. One reason for this is a lack of appreciation of what needs to be reproduced and how, in each phase of the innovation process. In the discovery phase, conclusions need to be reproduced through orthogonal investigation. In the translation phase, key attributes and outputs of derived products or processes should be reproducible by defining transferable specifications and protocols, whereas in the application phase, the goal is to achieve reproducible performance in real-world environments through appropriate quality assurance systems.

## Introduction

**Figure 1.**
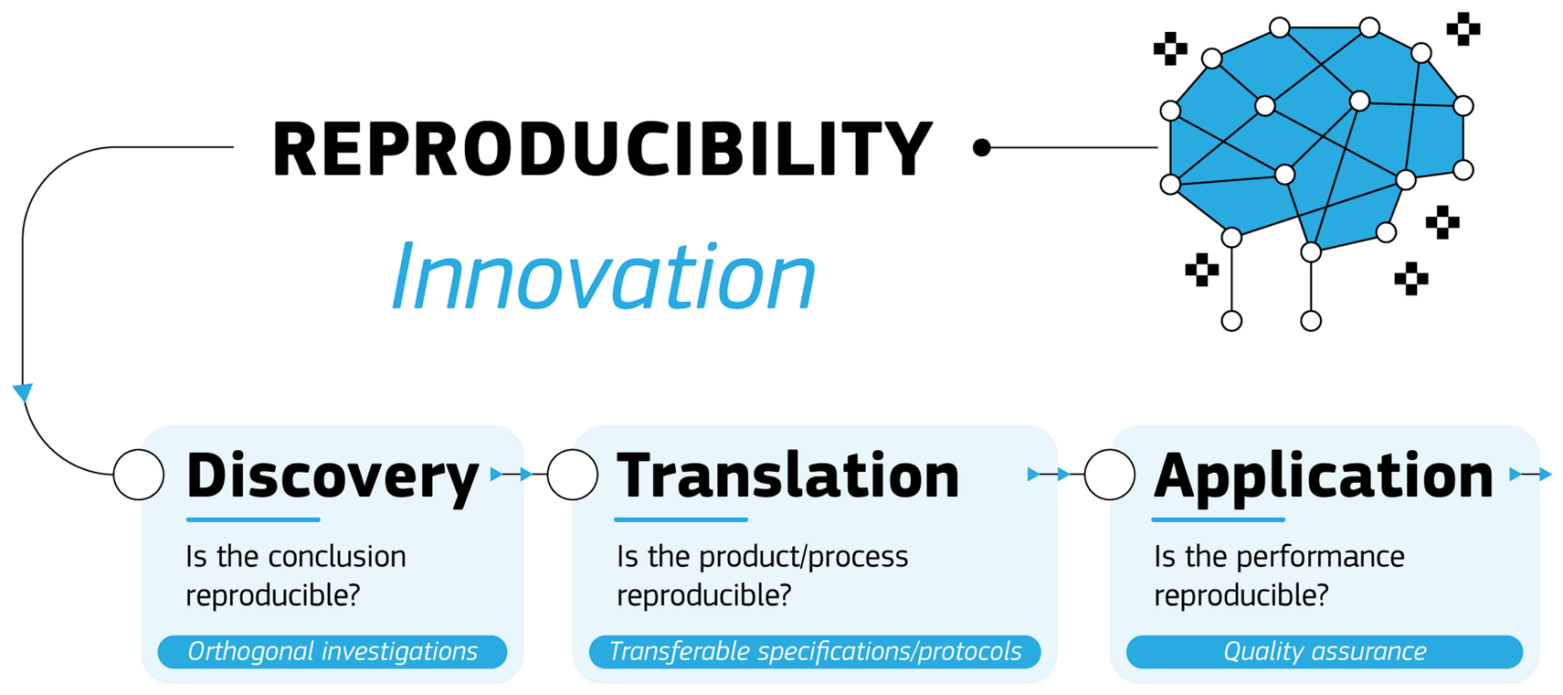
Schematic representation of the three phases of the innovation process and how to approach reproducibility in each.

It has been widely reported that there is a reproducibility crisis in science ^1– 4^ and that the economic and reputational costs of results which cannot be reproduced are likely far higher than the costs of repeating experiments ^5, 6^. Others see opportunity in embracing the challenges surrounding reproducibility, for better science teaching ^7^ and the creation of reinvigorated innovation ecosystems ^8^. There is much advice in the scientific literature about conducting studies that are more likely to be reproducible, most of which is based on three tenets: good study design based on a sound understanding of the underpinning science ^5^; robust methodology execution following the study design using appropriately calibrated and maintained equipment operated by trained personnel ^9^; and open, transparent reporting of study protocols, measurement procedures, data acquisition and analysis, including algorithms and codes ^10^. In addition to this however, as illustrated in Figure 1, we propose that the three major phases of the innovation process, namely discovery, translation and application, represent different contexts in which to consider reproducibility and thus a different approach is required for each.

The discovery phase typically involves demonstration of basic principles to proof-of-concept, representing a Technology Readiness Level (TRL) from 1–3 ^11^. In this initial phase, it is the reproducibility of the original conclusion that is paramount, which needs to be more than producing the same result in the same way. To provide sufficient confidence in a discovery for the innovation process to progress, it is necessary to arrive at the same conclusion via multiple orthogonal routes, i.e., independent demonstrations of the same conclusion. In other words, epistemic parity needs to be achieved by establishing that the same conclusion can be reached using a range of methodologies that generate their own appropriate data. Hence, it is important that new knowledge arising from the discovery is described in a manner that facilitates the design of appropriate orthogonal confirmatory routes. 

The translation phase of the innovation process is from laboratory demonstration of a prototype (TRL 4) to full-scale demonstration in an environment representing the real world (TRL 6). In this phase, the prototype product or process must be reproducible in terms of predefined attributes and outputs associated with fulfilling its purpose. For this, it is essential to have transferable specifications and protocols that allow prototype products and processes to be deployed in different representative environments by different users while achieving parity of operation and output. When describing an element of a specification or a step in a protocol therefore, the appropriate level of detail is needed, neither too little nor too much, together with the rationale for including particular elements or steps.

The final phase covers demonstration in the operational environment (TRL 7) of an operational product or process (TRL 9), termed the application phase. Here the aim is to achieve parity in overall performance. Customers expect reliability in products and processes, i.e., that they will perform in a consistent manner over time. This requires both robustness in the design of the final product or process and the implementation of appropriate quality assurance procedures so that the expected success rate is achieved across both production items and their lifecycle. Delivery of a consistent success rate is the form of reproducibility expected in the application phase of the innovation process. If an organisation fails to deliver this form of reproducibility, then it is unlikely to be commercially successful or viable.

It is usual for each of the three phases of the innovation process to be undertaken by separate groups of people, often in different organisations with differing motivations and incentives. Discovery generally occurs in research laboratories in universities or specialist research institutes where the drivers are advancing science, publishing scientific papers and attracting funding. Translation is often performed in applied research organisations that have a specific mission and setup to bridge the gap between discovery and application. The application phase is predominantly undertaken by commercial organisations when they see the potential of a new product or process to generate revenue and capture market share. In some scenarios these distinctions are blurred, for example when a university spinout company attempts to pursue the entire innovation process. While this blurring can avoid the creation of silos that can stall innovation, it also has the potential to cause a failure to recognise the different contexts of reproducibility in the phases of the innovation process.

In conclusion, successful innovation requires a better understanding and appreciation of the philosophical, contextual and practical differences in establishing reproducibility within the discovery, translation and application phases of the innovation process. This will improve the design, conduct and outcomes of reproducibility studies and facilitate more fruitful discussion and cooperation between key actors.

## Disclaimer

The views expressed in this article are those of the authors. Publication in Open Research Europe does not imply endorsement by the European Commission.

## Ethics and consent

Ethical approval and consent were not required.

## Data Availability

No data are associated with this article.

## References

[1] Prinz F Schlange T Asadullah K : Believe it or not: how much can we rely on published data on potential drug targets? *Nat Rev Drug Discov.* 2011;10(9): 712. 10.1038/nrd3439-c1 21892149

[2] Baker M : Reproducibility crisis. *Nature.* 2016;533(26):353–66.27193681

[3] Poldrack RA : The costs of reproducibility. *Neuron.* 2019;101(1):11–14. 10.1016/j.neuron.2018.11.030 30605654

[4] Camerer CF Dreber A Holzmeister F : Evaluating the replicability of social science experiments in nature and science between 2010 and 2015. *Nat Hum Behav.* 2018;2(9):637–644. 10.1038/s41562-018-0399-z 31346273

[5] Mann DL : The rising cost of developing cardiovascular therapies and reproducibility in translational research: do not blame it (all) on the bench. *JACC Basic Transl Sci.* 2017;2(5):627–629. 10.1016/j.jacbts.2017.09.006 30062177 PMC6058937

[6] Dirnagl U Duda GN Grainger DW : Reproducibility, relevance and reliability as barriers to efficient and credible biomedical technology translation. *Adv Drug Deliv Rev.* 2022;182: 114118. 10.1016/j.addr.2022.114118 35066104

[7] Karathanasis N Hwang D Heng V : Reproducibility efforts as a teaching tool: a pilot study. *PLoS Comput Biol.* 2022;18(11): e1010615. 10.1371/journal.pcbi.1010615 36355750 PMC9648701

[8] Munafò MR Chambers C Collins A : The reproducibility debate is an opportunity, not a crisis. *BMC Res Notes.* 2022;15(1):1–3, 43. 10.1186/s13104-022-05942-3 35144667 PMC8832688

[9] Lusoli W , (ed): Reproducibility of scientific results in the EU. European Commission, Directorate-General for Research and Innovation, Brussels,2020. 10.2777/341654

[10] Goodman SN Fanelli D Ioannidis JPA : What does research reproducibility mean? *Sci Transl Med.* 2016;8(341): 341ps12. 10.1126/scitranslmed.aaf5027 27252173

[11] Olechowski A Eppinger SD Joglekar N : Technology readiness levels at 40: a study of state-of-the-art use, challenges, and opportunities. In: *2015 Portland international conference on management of engineering and technology (PICMET).*IEEE,2015;2084–2094. 10.1109/PICMET.2015.7273196

