# Achieving reproducibility in the innovation process

**DOI:** 10.12688/openreseurope.19408.2

**Published:** 2025-06-30

**Authors:** Maurice Whelan, Eann Patterson

**Affiliations:** 1 European Commission Joint Research Centre (JRC), Ispra, 21027, Italy; 2 School of Engineering, University of Liverpool, Liverpool, L69 3GH, UK

**Keywords:** Innovation, reproducibility, discovery, translation, application

## Abstract

Reproducibility is essential for innovation but is often hard to achieve in practice. One reason for this is a lack of appreciation of what needs to be reproduced and how in each phase of the innovation process. In the discovery phase, conclusions need to be reproduced through orthogonal investigation. In the translation phase, key attributes and outputs of derived products or processes should be reproducible by defining transferable specifications and protocols, whereas in the application phase, the goal is to achieve reproducible performance in real-world environments through appropriate quality assurance systems.

## Introduction

It has been widely reported that there is a reproducibility crisis in science. In 2016 for example, Baker reported in a survey of 1575 scientists from biology, chemistry, earth & environment, medicine and physics & engineering, that most scientists had experienced a failure to reproduce results ^1^. In a very recent survey, Chakravorti *et al*. surveyed 452 professors in India and the USA across a wide spectrum of disciplines and described ‘national and disciplinary gaps in attention to reproducibility and transparency in science’ ^2^. There are reputational costs to both individuals and the scientific community ^3^ of results that cannot be reproduced, as well as economic costs such as in medicine when drug trials fail ^4, 5^. Conversely, a recent and extensive set of reproducibility and replicability studies in the Netherlands across social sciences, medical sciences and humanities, found that replication corroborates original findings, enhances understanding, and is an educational tool ^6^.

There is much confusion in the literature about the terms ‘reproducibility’ and ‘replicability’ with different disciplines adopting contradictory definitions, as discussed by Barba ^7^ and by Plesser ^8^, and more recently by Antunes & Hill ^9^ in context of high performance computing, who highlight that the Association of Computer Machinery (ACM) swapped their definitions of the terms in 2020. A dictionary definition of the adjective ‘reproducible’ is ‘ *that can be produced or done again in the same way*’ ^10^, while in metrology it is defined in terms of precision or closeness of agreement between replicate measurements ^11^. A 2015 report to the US National Science Foundation (NSF) ^12^ defined reproducibility as ‘the ability of a researcher to duplicate the results of a prior study using the same materials and procedures as were used by the original investigator’, which can be expressed as the same raw data and the same statistical analysis; while replicability refers to the ability of a researcher to ‘duplicate results of a prior study if the same procedures are followed but new data are collected’. Similar definitions were adopted by the US National Academies of Sciences, Engineering and Medicine in 2019 ^13^ and the ACM ^14^, although the latter’s definition of replicability is somewhat broader: ‘obtaining consistent results across studies aimed at answering the same question, each of which has obtained its own data’.

**Figure 1.**
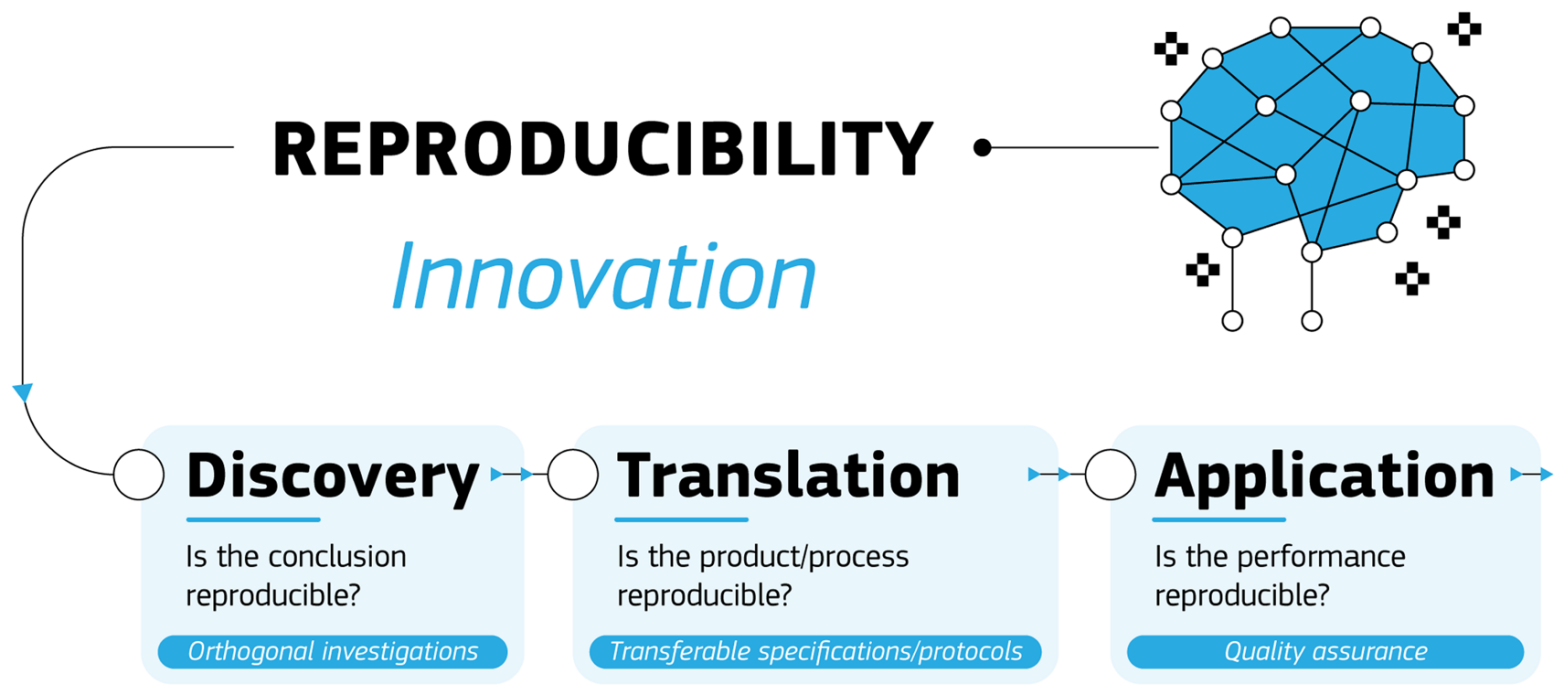
Schematic representation of the three phases of the innovation process and how to approach reproducibility in each.

Plenty of advice can be found in the scientific literature about conducting studies that are more likely to be reproducible, most of which is based on three tenets: good study design based on a sound understanding of the underpinning science ^5^; robust methodology execution following the study design using appropriately calibrated and maintained equipment operated by trained personnel ^15^; and open, transparent reporting of study protocols, measurement procedures, data acquisition and analysis, including algorithms and codes ^16^. However, most guidance, whilst sound, does not address the issues that a lack of reproducibility and replicability can create downstream in the innovation process. Hence, in addition to this guidance and in a novel departure, in this letter, we propose that the three major phases of the innovation process, namely discovery, translation and application, represent different contexts in which to consider reproducibility and replicability; and thus a different approach is required for each phase, as illustrated in Figure 1. Successful innovation, which is the practical implementation of ideas resulting in new or improved products or processes, requires a good understanding and appreciation of the challenges faced in each phase and thus the intent of our proposal is to stimulate productive discussion and interactions about these challenges between the stakeholders in any innovation process.

## Proposed approach

The discovery phase typically involves demonstration of basic principles to proof-of-concept, representing a Technology Readiness Level (TRL) from 1–3 ^17^. In this initial phase, it is the generalisation of the original conclusion that is paramount, which needs to be more than producing the same result in the same way by the same team (repeatable) or by a different team (reproducible). To provide sufficient confidence in a discovery for the innovation process to progress, it is necessary to arrive at the same conclusion via multiple orthogonal routes, i.e., independent demonstrations of the same conclusion. In other words, epistemic parity needs to be achieved by establishing that the same conclusion can be reached using a range of methodologies that generate their own appropriate data, which goes beyond replicability to ‘generalised’ outcomes. Hence, it is important that new knowledge arising from the discovery is described in a manner that facilitates the design of appropriate orthogonal confirmatory routes. 

The translation phase of the innovation process is from laboratory demonstration of a prototype (TRL 4) to full-scale demonstration in an environment representing the real world (TRL 6). In this phase, the prototype product or process must be reproducible in terms of predefined attributes and outputs associated with fulfilling its purpose. For this, it is essential to have transferable specifications and protocols that allow prototype products and processes to be deployed in different representative environments by different users while achieving parity of operation and output (different team, same setup = reproducibility). When describing an element of a specification or a step in a protocol therefore, the appropriate level of detail is needed, neither too little nor too much, together with the rationale for including particular elements or steps. In a commercial environment, reproducibility will need to be achieved within an organisation perhaps at different locations but probably not in different organisations, unless the intention is to licence the technology to other organisations. In some cases, the value of an organisation will be related to its ability to reliably reproduce prototype results for stakeholders, including potential investors. 

The final phase covers demonstration in the operational environment (TRL 7) of an operational product or process (TRL 9), termed the application phase. Here the aim is to achieve parity in overall performance. Customers expect reliability in products and processes, i.e., that they will perform in a consistent manner over time. This requires both robustness in the design of the final product or process and the implementation of appropriate quality assurance procedures so that the expected success rate is achieved across both production items and their lifecycle. Delivery of a consistent success rate is the form of reproducibility expected in the application phase of the innovation process (different team, same setup = reproducibility). If an organisation fails to deliver this form of reproducibility, then it is unlikely to be commercially successful or viable. At the same time, commercial success is likely to depend on protecting the intellectual property underpinning the innovation; hence, there is a balance to be achieved between achieving the expected reliability of products and preventing competitors from reproducing the same success. In this context, it is important to be able to define the attributes of reproducibility in order to be able to protect them.

It is usual for each of the three phases of the innovation process to be undertaken by separate groups of people, often in different organisations with differing motivations and incentives. Discovery generally occurs in research laboratories in universities or specialist research institutes where the drivers are advancing science, publishing scientific papers and attracting funding. Translation is often performed in applied research organisations that have a specific mission and setup to bridge the gap between discovery and application. The application phase is predominantly undertaken by commercial organisations when they see the potential of a new product or process to generate revenue and capture market share. In some scenarios these distinctions are blurred, for example when a university spinout company attempts to pursue the entire innovation process. While this blurring can avoid the creation of silos that can stall innovation, it also has the potential to cause a failure to recognise the different contexts of reproducibility in the phases of the innovation process.

In conclusion, successful innovation requires a better understanding and appreciation of the philosophical, contextual and practical differences in establishing reproducibility within the discovery, translation and application phases of the innovation process. This will improve the design, conduct and outcomes of reproducibility studies and facilitate more fruitful discussion and cooperation between key actors.

## Disclaimer

The views expressed in this article are those of the authors. Publication in Open Research Europe does not imply endorsement by the European Commission.

## Ethics and consent

Ethical approval and consent were not required.

## Data Availability

No data are associated with this article.

## References

[1] Baker M : Reproducibility crisis. *Nature.* 2016;533(26):353–66.27193681

[2] Chakravorti T Koneru S Rajtmajer S : Reproducibility and replicability in research: what 452 professors think in Universities across the USA and India. *PLoS One.* 2025;20(3): e0319334. 10.1371/journal.pone.0319334 40138274 PMC11940819

[3] Hagiopol C Leru PM : Scientific truth in a post-truth era: a review. *Sci & Educ.* 2024;1–34. 10.1007/s11191-024-00527-x

[4] Prinz F Schlange T Asadullah K : Believe it or not: how much can we rely on published data on potential drug targets? *Nat Rev Drug Discov.* 2011;10(9):712. 10.1038/nrd3439-c1 21892149

[5] Mann DL : The rising cost of developing cardiovascular therapies and reproducibility in translational research: do not blame it (all) on the bench. *JACC Basic Transl Sci.* 2017;2(5):627–629. 10.1016/j.jacbts.2017.09.006 30062177 PMC6058937

[6] Derksen M Meirmans S Brenninkmeijer J : Replication studies in the Netherlands: lessons learned and recommendations for funders, publishers and editors, and universities. *Account Res.* 2024;1–19. 10.1080/08989621.2024.2383349 39135508

[7] Barba LA : Terminologies for reproducible research. *arXiv preprint arXiv:* 1802.03311.2018. 10.48550/arXiv.1802.03311

[8] Plesser HE : Reproducibility vs. replicability: a brief history of a confused terminology. *Front Neuroinform.* 2018;11: 76. 10.3389/fninf.2017.00076 29403370 PMC5778115

[9] Antunes B Hill DRC : Reproducibility, replicability and repeatability: a survey of reproducible research with a focus on high performance computing. *Comput Sci Rev.* 2024;53: 100655. 10.1016/j.cosrev.2024.100655

[10] Oxford english dictionary. J Simpson & E Wiener (eds): 2 ^nd^Edition, Oxford: Oxford University Press,1989. Reference Source

[11] Joint Committee for Guides in Metrology: International vocabulary of metrology—basic and general concepts and associated terms. Paris, France: International Organization of Legal Metrology,2006. Reference Source

[12] Bollen K Cacioppo JT Kaplan R : Social, behavioral, and economic sciences perspectives on robust and reliable science. National Science Foundation, Arlington, VA,2015. Reference Source

[13] Committee on Reproducibility and Replicability in Science: Reproducibility and replicability in science.2019; last accessed June 11 ^th^, 2025. 10.17226/25303 31596559

[14] 2024 Association of Computer Machinery Conference on Reproducibility and Replicability. last accessed 11 ^th^June 2025. Reference Source

[15] Lusoli W , (ed): Reproducibility of scientific results in the EU.European Commission, Directorate-General for Research and Innovation, Brussels,2020. 10.2777/341654

[16] Goodman SN Fanelli D Ioannidis JPA : What does research reproducibility mean? *Sci Transl Med.* 2016;8(341): 341ps12. 10.1126/scitranslmed.aaf5027 27252173

[17] Olechowski A Eppinger SD Joglekar N : Technology readiness levels at 40: a study of state-of-the-art use, challenges, and opportunities.In: *2015 Portland international conference on management of engineering and technology (PICMET)*. IEEE, August,2015;2084–2094. 10.1109/PICMET.2015.7273196

