# Supplementary material for: Achieving reproducibility in the innovation process
Source: Open Res Eur. 2025 Jun 30;5:25. Originally published 2025 Jan 27. [Version 2] doi: 10.12688/openreseurope.19408.2 (PMC12397739; doi:10.12688/openreseurope.19408.2)
Supplement: Peer review history

# Reviewer report: Reviewer response for version 2

**DOI:** 10.21956/openreseurope.22484.r56904

**Published:** 2025-09-01

**Authors:** Yibei Chen

**Affiliations:** 1 Massachusetts Institute of Technology, Cambridge, USA

This paper starts with the reproducibility crisis in science and then argues that the practices recommended for improving reproducibility in science do not apply to the innovation process, which is essentially the industrialization of scientific products. This is an interesting perspective. However, it also implies that the transition from science to innovation/technology may only apply to certain types of science.

 The paper then proposes three phases: (1) demonstration of basic principles to proof-of-concept (TRL 1–3), (2) translation of lab prototype (TRL 4) to full-scale demonstration in the real world (TRL 6), and (3) demonstration in the operational environment (TRL 7) of an operational product or process (TRL 9).

 But what about TRL 5 and TRL 8? Are they intermediate steps between 4 and 6, and 7 and 9?

 I’m not familiar with the industry context, but here are some comments that may help make the article more accessible to readers:

 1. If the goal of this paper is to propose how to achieve reproducibility in the innovation process, the introduction should start with the core concepts, “reproducibility” and “innovation process”, rather than focusing first on reproducibility and replicability in science. The opening paragraph could go straight to the core argument instead of providing such a broad context.

 2. In the “Proposed approach” section, the three phases are quite abstract. It would help if the author provided examples to illustrate them. This could be done with one example that moves through all three phases, or three separate examples, one for each phase.

 3. It would also be helpful if the paper included a clear, concrete definition of Technology Readiness Levels, ideally covering TRL 1 through 9, so readers can see the full picture.

 4. “Achieving reproducibility” in the three phases remains quite conceptual. The paper could be strengthened by including operationalized definitions along with examples. A table could work well here, listing: (1) Rules/advice for achieving reproducibility, (2) Conceptual definition, (3) Operationalized definition, and (4) Real-world example

Where applicable, are recommendations and next steps explained clearly for others to follow? (Please consider whether others in the research community would be able to implement guidelines or recommendations and/or constructively engage in the debate)

Partly

Does the article adequately reference differing views and opinions?

Partly

Are all factual statements correct, and are statements and arguments made adequately supported by citations?

Yes

Is the rationale for the Open Letter provided in sufficient detail? (Please consider whether existing challenges in the field are outlined clearly and whether the purpose of the letter is explained)

Partly

Is the Open Letter written in accessible language? (Please consider whether all subject-specific terms, concepts and abbreviations are explained)

Yes

Reviewer Expertise:

neuroscience

I confirm that I have read this submission and believe that I have an appropriate level of expertise to confirm that it is of an acceptable scientific standard, however I have significant reservations, as outlined above.

# Reviewer report: Reviewer response for version 2

**DOI:** 10.21956/openreseurope.22484.r56902

**Published:** 2025-08-29

**Authors:** Joris Frese

**Affiliations:** 1 European University Institute, Florence, Italy

In this open letter, the authors combine discussions about reproducibility/replicability in science with discussions about the innovation process in industrial settings. The key claim that the paper argues for is that there are three distinct phases in the innovation process of companies (discovery, translation, application), that each require distinct approaches to reproducibility. The authors propose ways to take reproducibility into account during each phase.

 While I am not an expert whatsoever on industrial innovation processes (my expertise is rather related to replicability in scientific research), I appreciate the attempt to use this scientific lingo and transfer it to other areas. The authors´ argument that reproducibility is important yet underappreciated in industry lingo seems plausible to me and if that is indeed the case, then their letter makes a valuable contribution.

 I also appreciate that the authors addressed various comments made by the previous reviewers compared to version 1 of this letter. I do however think that two points of the previous reviewers (which I agree with) have not yet been sufficiently addressed:

 1) As mentioned in a previous review, there is quite an abrupt switch of focus from the introduction to the main text. First, the authors talk almost exclusively about reproducibility in scientific research, but then the last paragraph of the introduction and the main text are suddenly adopting a very industry/engineering-focussed perspective. I believe it might be worthwhile to add another paragraph in the introduction that clearly lays out the benefits of adopting reproducibility-lingo from science/academia to problems in industry/engineering/innovation enterprises and then keep the rest of the letter focussed on those latter aspects, rather than seemingly being stuck in the twilight zone between industry and academia (I believe academia already has more than its fair share of opinion pieces on the reproducibility crisis, so a piece focussed primarily and clearly on reproducibility issues in industry might be more valuable).

 2) As also mentioned in a previous review, the letter would benefit substantially from incorporating some more concrete examples into the main text (e.g., what is one concrete example of a situation where companies need to worry about reproduciblity during the translation and/or the application phase of the innovation process). Keeping everything at the rather abstract level that it is currently at might risk confusing many non-expert readers who could otherwise benefit from this piece.

Where applicable, are recommendations and next steps explained clearly for others to follow? (Please consider whether others in the research community would be able to implement guidelines or recommendations and/or constructively engage in the debate)

Partly

Does the article adequately reference differing views and opinions?

Yes

Are all factual statements correct, and are statements and arguments made adequately supported by citations?

Yes

Is the rationale for the Open Letter provided in sufficient detail? (Please consider whether existing challenges in the field are outlined clearly and whether the purpose of the letter is explained)

Yes

Is the Open Letter written in accessible language? (Please consider whether all subject-specific terms, concepts and abbreviations are explained)

Partly

Reviewer Expertise:

metascience; open science; reproducibility; interdisciplinary collaboration

I confirm that I have read this submission and believe that I have an appropriate level of expertise to confirm that it is of an acceptable scientific standard, however I have significant reservations, as outlined above.

# Reviewer report: Reviewer response for version 2

**DOI:** 10.21956/openreseurope.22484.r56277

**Published:** 2025-08-21

**Authors:** Tim Booth

**Affiliations:** 1 DTU, Kongens Lyngby, Denmark

The revised manuscript addresses the majority of the concerns raised in my initial review.

 The authors have clarified their intent and strengthened the philosophical and contextual framing of reproducibility across the three proposed phases of the innovation process. The addition of a paragraph discussing the divergent uses of "reproducibility," "replicability," and "repeatability" across disciplines, including references to Barba, Plesser, and Antunes & Hill, significantly improves the manuscript’s clarity and positioning. Their choice to retain a broad, epistemological use of "reproducibility" is now more clearly justified and appropriately caveated.

 Further, the discussion of the translation and application phases has been expanded and sharpened. The authors now acknowledge that reproducibility in these contexts may be internal to organisations and shaped by commercial concerns such as IP protection and stakeholder confidence. This framing is more useful and realistic.

 The reference base has been broadened and now better reflects the interdisciplinary nature of the topic, though life sciences still dominate the examples.

 That said, one point remains underdeveloped. While the manuscript now does a good job of outlining *why* reproducibility takes different forms in discovery, translation, and application, it still leaves open the question of *what should be done differently*. Having mapped reproducibility needs onto TRL stages and organizational types, the logical next step would be to indicate how stakeholders in each context might act on these insights. What should academic researchers working in early-stage discovery consider when designing reproducible claims for downstream translation? What protocols or specification strategies might applied research centers adopt to enable transfer? How can quality assurance in the application phase be framed not only as a technical obligation but as a communicable standard for investors or regulatory bodies? Even brief examples or references to existing practices would help anchor the authors’ framework in practical decision-making. As it stands, the manuscript remains largely conceptual, and readers looking for guidance may be left unsure of how to act on the insights presented by the end of the article.

 Finally, I would note that a means of identifying the changes in the revised manuscript would have been greatly appreciated. As it stands, there is no documentation of the edits, and readers are left to manually compare versions. A brief point-by-point summary or marked-up copy would have facilitated a more efficient review process.

 In summary, the revisions significantly improve the manuscript, and I would now consider it suitable for indexing, with the noted reservations.

Where applicable, are recommendations and next steps explained clearly for others to follow? (Please consider whether others in the research community would be able to implement guidelines or recommendations and/or constructively engage in the debate)

Partly

Does the article adequately reference differing views and opinions?

Partly

Are all factual statements correct, and are statements and arguments made adequately supported by citations?

Partly

Is the rationale for the Open Letter provided in sufficient detail? (Please consider whether existing challenges in the field are outlined clearly and whether the purpose of the letter is explained)

Yes

Is the Open Letter written in accessible language? (Please consider whether all subject-specific terms, concepts and abbreviations are explained)

Yes

Reviewer Expertise:

2D Materials, Innovation

I confirm that I have read this submission and believe that I have an appropriate level of expertise to confirm that it is of an acceptable scientific standard, however I have significant reservations, as outlined above.

# Reviewer report: Reviewer response for version 2

**DOI:** 10.21956/openreseurope.22484.r56276

**Published:** 2025-08-21

**Authors:** Antunes Benjamin

**Affiliations:** 1 Universite Clermont Auvergne, Clermont-Ferrand, France; 2 University of Perpignan Via Domitia (Ringgold ID: 27074), Perpignan, Occitanie, France

The added section about reproducible research terminology, and the correct usage of the different terms (repeatability, reproducibility, and replicability), makes the paper clearer.

 We can now better see the connection between TRL terminology used in industry and the concept of reproducibility.

 The authors highlighted the importance of the reproducibility process for industry, while acknowledging that it can vary depending on intellectual property. In my opinion, the different contexts are now well defined.

Where applicable, are recommendations and next steps explained clearly for others to follow? (Please consider whether others in the research community would be able to implement guidelines or recommendations and/or constructively engage in the debate)

Not applicable

Does the article adequately reference differing views and opinions?

No

Are all factual statements correct, and are statements and arguments made adequately supported by citations?

Partly

Is the rationale for the Open Letter provided in sufficient detail? (Please consider whether existing challenges in the field are outlined clearly and whether the purpose of the letter is explained)

Partly

Is the Open Letter written in accessible language? (Please consider whether all subject-specific terms, concepts and abbreviations are explained)

Yes

Reviewer Expertise:

Reproducibility in computer science

I confirm that I have read this submission and believe that I have an appropriate level of expertise to confirm that it is of an acceptable scientific standard.

# Reviewer report: Reviewer response for version 1

**DOI:** 10.21956/openreseurope.21002.r53244

**Published:** 2025-05-19

**Authors:** Tim Booth

**Affiliations:** 1 DTU, Kongens Lyngby, Denmark

The authors argue that reproducibility, a cornerstone of scientific and technological credibility, must be understood in relation to three distinct phases of the innovation process: i) Discovery, ii) Translation, and iii) Application. Each phase is associated with different reproducibility criteria: epistemic confirmation for discovery, specification transferability for translation, and performance consistency for application. These phases are mapped to Technology Readiness Levels (TRLs), offering a structured lens to evaluate reproducibility across the innovation lifecycle.

 The conceptual framing of reproducibility in innovation in terms of these three phases is a thoughtful attempt to bridge epistemological concerns from academia with operational needs in industry. This appears to be a novel and potentially impactful contribution, particularly for research managers and policymakers. However, the letter does not sufficiently distinguish itself from prior work on the reproducibility crisis in academic contexts, nor does it fully resolve tensions between academic and industrial interpretations of reproducibility.

 Data and methodology

 This is a conceptual piece without original data, but the reasoning is generally coherent. However, the proposal would strongly benefit from concrete examples or case studies illustrating how reproducibility efforts differ (or should differ) across TRLs.

 Terminology: The authors' use of "reproducibility" to cover what is elsewhere called "replicability" or "repeatability" may confuse readers, especially in computational and biomedical domains where these terms have distinct meanings. While some justification is provided through epistemological framing, a clearer acknowledgment of prior work that sharpens the definition of competing terminologies (e.g. refer 1, refer 2) would improve clarity and credibility. Readers may disagree that confirmation of conclusions through orthogonal experimental routes qualifies as reproducibility. Conversely, if one cannot independently verify a conclusion using different methods, samples, and data, it might be difficult to argue that the original conclusion is not reproducible in a precise sense, rather than irreplaceable in the current investigation. The paper would benefit from a more explicit justification of the terminology used, particularly the umbrella use of "reproducibility" in light of current consensus in relevant fields such as computational science, engineering, and biology.

 Further, if the aim is to influence innovation practice, the authors should enrich the article by proposing concrete tools or mechanisms (for example, protocol repositories, QA systems, or documentation practices) that could enhance reproducibility at each phase. This would allow stakeholders to more easily action the authors' recommendations.

 Many references are drawn from biomedical sciences. Inclusion of examples from materials science, computer science, or engineering would help the piece resonate across more disciplines. While biomedical sciences have naturally dominated the literature on reproducibility due to high stakes, system complexity, and challenges in methodological transparency, there is a wide acknowledgement of the problem across fields.

 In their effort to encompass and speak to as many stakeholders as possible, the authors risk conflating issues in reproducibility of knowledge generation (academic) with those of production and deployment (industrial). The three-phase breakdown used by the authors is a positive step, though arguably, it is the challenges in the Discovery phase that lie at the heart of most reproducibility concerns. The Translation and Application steps, for example, rarely require communication of protocols to other stakeholders for reasons of industrial competition and secrecy. It is difficult to imagine start-ups spending much time assisting other start-ups in reproducing their competitive advantage, or incumbent industry leaders helping competitors achieve parity. The authors might assist readers by clarifying the specific issues in phases ii) and iii).

 Several minor language issues (such as missing commas or slightly awkward constructions) could be addressed with a careful copy-edit.

 Conclusions

 The article makes a worthwhile conceptual contribution by linking reproducibility concerns to the innovation pipeline and suggesting that different forms of reproducibility are relevant at different stages. However, in its current form, the piece straddles the boundary between commentary and actionable guidance without fully satisfying either mode. With the addition of clearer definitions, concrete examples, and interdisciplinary breadth, the article could be a useful resource for those involved in translational research, policy, and innovation governance.

 Recommendation

 Major revision. The core idea is solid but requires clarification, expansion, and sharper framing to fully realize its potential contribution.

Where applicable, are recommendations and next steps explained clearly for others to follow? (Please consider whether others in the research community would be able to implement guidelines or recommendations and/or constructively engage in the debate)

Partly

Does the article adequately reference differing views and opinions?

Partly

Are all factual statements correct, and are statements and arguments made adequately supported by citations?

Partly

Is the rationale for the Open Letter provided in sufficient detail? (Please consider whether existing challenges in the field are outlined clearly and whether the purpose of the letter is explained)

Yes

Is the Open Letter written in accessible language? (Please consider whether all subject-specific terms, concepts and abbreviations are explained)

Yes

Reviewer Expertise:

2D Materials, Innovation

I confirm that I have read this submission and believe that I have an appropriate level of expertise to confirm that it is of an acceptable scientific standard, however I have significant reservations, as outlined above.

## References

[1] https://arxiv.org/abs/1802.03311.

[2] https://doi.org/10.1016/j.cosrev.2024.100655.

# Reviewer report: Reviewer response for version 1

**DOI:** 10.21956/openreseurope.21002.r50404

**Published:** 2025-02-19

**Authors:** Antunes Benjamin

**Affiliations:** 1 Universite Clermont Auvergne, Clermont-Ferrand, France; 2 University of Perpignan Via Domitia (Ringgold ID: 27074), Perpignan, Occitanie, France

This open letter discusses reproducibility in science. The paper proposes that the innovation process is divided into three categories: discovery, translation, and application. It states that reproducibility concerns vary across these categories.

 For the first category, the focus is on the reproducibility of scientific conclusions. For the second, it concerns the reproducibility of the protocols and processes used to develop the product. For the third, it addresses the reproducibility of the final quality of the product, which is essential for commercial success.

 For all these stages, they use the “Technology Readiness Level” (TRL) scale, that is a “tool for assessing the maturity of technologies during complex system development” to measure the maturity of the project.

My comments:

 The introduction of this paper begins by presenting the challenges we face regarding the reproducibility of published research papers. In my opinion, the references cited [1-10] primarily address academic research, particularly the lack of reproducibility in published studies (due to multiple reasons, such as not sharing the data, the code, bad statistics, …). The paper also provides some clarification on three key tenets they consider essential for reproducibility: good study design, robust methodological execution, and open science, which includes sharing code, data, etc.

 However, it seems that the authors then shift their focus to industrial processes rather than academic research. In my view, concerns about reproducibility in industry differ from those in academic research. In science (academia), our objective is to build knowledge upon the existing knowledge of others. The reproducibility crisis mentioned in the introduction refers to this issue: if research papers cannot be reproduced, it raises concerns about the reliability of published results and our ability to build upon previous research.

 In an industrial context, however, a probable goal of the company is growth and profitability. Of course, standardizing the products they sell is essential (as discussed in the "application" category), but can we truly talk about reproducibility in this context? A company may aim for internal reproducibility, but its objective might not be to make its protocols and processes (category “translation”) or its final quality assurance (category “application”) easily reproducible by others.

 Therefore, in my opinion, this paper might be mixing these two distinct concepts: the reproducibility crisis in academic research (which pertains to scientific publications) and the industrial processes a company establishes to produce and sell standardized products. I fail to see the relevance of considering the latter in this discussion in the context of reproducibility.

 A scientific publication is an innovation in itself; it does not necessarily have to lead to the commercialization of a new product. In practice, only a very small percentage of scientific publications result in the production and commercialization of new products.

More focused comments:

 The paper uses the TRL scale to describe the maturity of a project. While this may be well-suited for industrial projects, I am unsure whether it effectively applies to scientific publications. The discovery phase corresponds to TRL levels 1 to 3, which include: *Basic principles observed and reported*, *Technology concept and/or application formulated* and *Analytical and experimental critical function and/or characteristic proof-of-concept*.

 The paper states “ *In this initial phase, it is the reproducibility of the original conclusion that is paramount, which needs to be more than producing the same result in the same way*”.  

 First, in my opinion, a published paper is a complete entity in itself. The process of reaching a conclusion is not limited to TRL levels 1 to 3. The tools and methods used—such as code, data, and other resources—significantly influence the final result and, consequently, the conclusion. I believe that a scientific publication is a project/innovation on its own, and this is where the reproducibility crisis lies, as well as the reproducibility issues highlighted in the references cited by the authors.

 Secondly, in the context of scientific research, the current consensus is not to use the term “reproducibility” when referring to obtaining the same scientific conclusion using a different method. In this context, the appropriate term is “replicability.” However, I acknowledge that in epistemology, "reproducibility" remains the broader term. Here are some references:

 BARBA, Lorena A. Terminologies for reproducible research. arXiv preprint arXiv:1802.03311, 2018[Ref-2].

 ANTUNES, Benjamin and HILL, David R.C. Reproducibility, Replicability and Repeatability: A survey of reproducible research with a focus on high performance computing, Computer Science Review, Volume 53, 2024 [Ref-2], 100655, ISSN 1574-0137, https://doi.org/10.1016/j.cosrev.2024.100655.

https://www.acm.org/publications/policies/artifact-review-and-badging-current

 “ *As a result of discussions with the National Information Standards Organization (NISO), it was recommended that ACM harmonize its terminology and definitions with those used in the broader scientific research community, and ACM agreed with NISO’s recommendation to swap the terms “reproducibility” and “replication” with the existing definitions used by ACM as part of its artifact review and badging initiative. ACM took action to update all prior badging to ensure consistency*”

Conclusion:

 Overall, in conclusion, I feel that I did not fully grasp the purpose of the article. Perhaps the issue is on my side. I believe that both the references cited and my own perspective focus more on the reproducibility of scientific research papers—whether academic or industry-based—, due to lack of data, code, and so on, while this article seems to address the reproducibility of industrial processes for producing a final commercial product.

 However, in this context, no specific tools are proposed. I am not an expert of industry, but considering the concept of reproducibility (if the entity producing and selling the final product wants everyone to be able to reproduce it), the authors could have suggested tools for different phases. For the discovery phase, they could have introduced tools for sharing code, data, or documentation methods. For the translation phase, maybe some workflow tools that facilitate process replication could have been discussed. For the application phase, maybe standardized quality assurance methods might have been relevant.

 Additionally, for a general-purpose paper on reproducibility, the references seem overly concentrated on biology (or related fields).

 Maybe I didn't grasp the interest of this publication. I am open for discussion.

Some typos:

 Abstract:

 Missing comma between “how” and “in”: One reason for this is a lack of appreciation of what needs to be reproduced and how in each phase of the innovation process.

 Introduction:

 Miss comma between “this” and “however”: In addition to this however, as illustrated

Where applicable, are recommendations and next steps explained clearly for others to follow? (Please consider whether others in the research community would be able to implement guidelines or recommendations and/or constructively engage in the debate)

Not applicable

Does the article adequately reference differing views and opinions?

No

Are all factual statements correct, and are statements and arguments made adequately supported by citations?

Partly

Is the rationale for the Open Letter provided in sufficient detail? (Please consider whether existing challenges in the field are outlined clearly and whether the purpose of the letter is explained)

Partly

Is the Open Letter written in accessible language? (Please consider whether all subject-specific terms, concepts and abbreviations are explained)

Yes

Reviewer Expertise:

Reproducibility in computer science

I confirm that I have read this submission and believe that I have an appropriate level of expertise to state that I do not consider it to be of an acceptable scientific standard, for reasons outlined above.

## References

[1] : Reproducibility, Replicability and Repeatability: A survey of reproducible research with a focus on high performance computing. *Computer Science Review* .2024;53: 10.1016/j.cosrev.2024.100655 10.1016/j.cosrev.2024.100655

[2] : Terminologies for Reproducible Research.2018; Reference source
